# Adiponectin gene polymorphisms rs1501299 and rs17300539 are not associated with polycystic ovary syndrome

**DOI:** 10.1590/1806-9282.20250864

**Published:** 2025-12-15

**Authors:** Emre Taşkin, Semra Eroğlu

**Affiliations:** 1 Bandırma Onyedi Eylül University, Faculty of Medicine, Department of Medical Genetics – Bandırma, Turkey.; 2 Samsun University, Samsun Practice Hospital, Faculty of Medicine, Department of Obstetrics and Gynecology – Samsun, Turkey.

**Keywords:** PCOS, Adiponectin, Polymorphism, Testosterone, Progesterone

## Abstract

**OBJECTIVE:**

Polycystic ovary syndrome is a common and important disease that affects ovarian functions in women of reproductive age. Disease—genetic variation associations between polymorphisms of the ADIPOQ gene rs1501299 and rs17300539, and polycystic ovary syndrome were studied in several populations previously and obtained divergent results; we have investigated this association in a case-control study for the first time in the studied population.

**METHODS:**

Forty-five polycystic ovary syndrome patients and 45 control participants were enrolled. Deoxyribonucleic acid of the participants was extracted from peripheral blood. Genotyping of subjects was made according to the real-time polymerase chain reaction method using custom primers. Allele and genotype frequencies, as well as clinical characteristics, were compared between the polycystic ovary syndrome and control groups using appropriate statistical methods.

**RESULTS:**

The mean testosterone level of the polycystic ovary syndrome group was significantly higher than the control group after multiple testing with Bonferroni correction (p<0.001). The mean progesterone level of the control group was significantly higher than the polycystic ovary syndrome group after multiple testing with Bonferroni correction (p<0.001). There were no significant differences between groups in terms of both allele and genotype frequencies (p>0.05). According to regression analysis, neither polymorphism had any effect on having polycystic ovary syndrome (p>0.05).

**CONCLUSION:**

For the first time in a sample from the studied ethnicity, we conclude that both rs1501299 and rs17300539 polymorphisms are not associated with polycystic ovary syndrome. Testosterone and progesterone levels were significantly affected by polymorphisms.

## INTRODUCTION

Polycystic ovary syndrome (PCOS) is a frequently seen disease of reproductive-aged women^1^. Prominent clinical manifestations are anovulation (reduced ovulation in some cases), multiple ovarian cysts, and hyperandrogenism, with occasional conditions like insulin resistance (IR) as well as type 2 diabetes (T2DM)^2^.

As a group of cytokines, adipokines are being secreted mainly from adipose tissue and take place in regulating energy metabolism and actions of anti-inflammation as well as decomposing fatty acids and adjusting glucose levels, which may be related to PCOS. Heterogeneity of PCOS makes it challenging to specify a precise genetic background to elucidate its etiology. Thus, investigating whether ADIPOQ gene polymorphisms are associated with PCOS in a population from Turkey for the first time may add useful information about the incidence and pathophysiology of the disease.

## METHODS

### Study population and participants

Forty-five PCOS patients and 45 control participants were recruited, who were admitted to the outpatient obstetrics and gynecology department of Karabuk University, Education and Research hospital of Medical Faculty. Patients were diagnosed with PCOS if their ultrasound images had two of the Rotterdam criteria: Oligo and/or anovulation, and polycystic ovaries. Phenotypes of the PCOS patients were characterized by ovulatory dysfunction and polycystic ovaries.

Exclusion criteria were being relatives and having any other metabolic or chronic diseases, as well as taking medications that may affect clinical measurements. Inclusion criteria were being diagnosed with PCOS for the PCOS group and being healthy participants aged between 17 and 42 for the control group. Before sample blood drawing, all participants of both case and control groups were verbally informed about the procedure of how the blood will be drawn, stored, and processed, as well as what kind of information will be extracted and processed. After the verbal consent, written and signed consent was also taken that all procedures were written on.

### Sample collecting and genotyping

After overnight fasting, 2 mL of venous blood was drawn into ethylenediaminetetraacetic acid-containing tubes. On the same day, 150 μL of genomic deoxyribonucleic acid (DNA) was extracted using a proper kit (NucleoSpin Blood, Mini kit for DNA from blood, Macherey-Nagel, Germany). Extracted DNA was stored at −20°C. Primer sequences for rs1501299 were forward 3′-TGTGTCTAGGCCTTAGTTAATAATGAATTC-5′ and reverse 3′-CTGTGTCTAGGCCTTAGTTAATAATGAATTA-5′, while forward 3′-AGAATGTGTGGCTTGCAAGAACTG-5′ and reverse 3′-CAGAATGTGTGGCTTGCAAGAACTA-5′. The Ct threshold method using cybergreen dye was used for genotyping. A total of 20 μL of polymerase chain reaction reaction mix was prepared for all polymorphisms. The ABI 7500 real-time system and software were used for amplification and detection of polymorphisms (Applied Biosystems). For hormone and biochemical measurements, the Siemens ADVIA Centaur^®^ XP and 2400 Clinical Chemistry System were used.

### Statistical analysis

For statistical analysis, SPSS software was used (IBM^®^). The chi-square method was used for checking the Hardy-Weinberg equilibrium, and no significant deviation was found in both the PCOS and the control groups. Variables for all groups were checked for normal distribution using the Kolmogorov-Smirnov test. Student's t-test was performed for normally distributed continuous variables. For non-normally distributed variables, the Mann-Whitney test was used. Genotype and allele frequency distribution between groups were checked with the chi-square test. Differences in clinical data were detected with the analysis of variance test. Where clinical data didn't follow normal distribution, the Kruskal-Wallis method was used for differences between genotypes. The Bonferroni method as a multiple testing correction was applied in order to avoid an elevated risk of false-positive findings. For the effect of polymorphisms on appearing in PCOS or control groups, a logistic regression test was used. Power and effect size calculations were done using G*Power software. The alpha level for significance was determined as p=0.05. Continuous variables were given as mean and standard deviation (SD).

## RESULTS

In Table 1 clinical characteristics of all participants were given. The mean testosterone level of the PCOS group was higher than the controls after Bonferroni correction (p<0.001). The mean progesterone levels were higher in the control group than the PCOS group after Bonferroni correction (p<0.001).

**Table 1:** Clinical data of all subjects.

Clinical data	Control group (n=45)	PCOS group (n=45)	p
Age (years)	26.1±7.1	23.7±5.8	>0.05
BMI (kg/m^2^)	24.9±3.5	24.1±5.4	>0.05
Fasting glucose (mmol/L)	5.14±0.81	5.14±0.61	>0.05
Insulin (pmol/L)	159±115	160±166	>0.05
Testosterone (nmol/L)	1.3±0.72	1.82±0.78	**<0.001**
Estradiol (pmol/L)	338±269	223±137	**=0.009**
Progesterone (ng/mL) (nmol/L)	15.6±13.1	3.88±7.8	**<0.001**
FSH (IU/L)	8.98±4.96	7.03±2.12	>0.05
LH (IU/L)	11.4±14.7	11.2±7.9	>0.05
Prolactin (μg/L)	11.9±8.02	15.77±10.5	**=0.05**
TSH (IU/L)	2.51±1.89	2.8±1.55	>0.05

BMI: body mass index; FSH: follicle-stimulating hormone; LH: luteinizing hormone; TSH: thyroid-stimulating hormone; PCOS: polycystic ovary syndrome. Statistically significant values are indicated in bold.

Table 2 shows regression results combined with genotype and allele frequencies for the sake of better readability. Neither polymorphism had any influence on having PCOS under the unadjusted and adjusted models, nor was there any difference between groups in terms of both genotype and allele frequencies (p>0.05).

**Table 2:** Regression results with genotype and allele frequencies.

RS1501299		Unadjusted model		Adjusted model^a^	
		OR (95%CI)	p	OR (95%CI)	p
Additive model regression	GG	1		1	
	GT	1.471 (0.614–3.524)	0.39	1.723 (0.684–4.339)	=0.25
	TT	0.706 (0.147–3.395)	0.67	0.893 (0.176–4.527)	=0.89
Dominant model regression	GG	1		1	
	GT+TT	1.318 (0.568–3.058)	0.52	1.554 (0.640–3.773)	=0.33
		**Controls (n=45)**	**PCOS (n=45)**		**p**
Genotype frequency comparison	GG	20 (44)	17 (38)		
	GT	20 (44)	25 (55)		
	TT	5 (12)	3 (7)		>0.05
Allele frequency	G/T	0.67/0.33	0.66/0.34		>0.05
Genotype frequency comparison	GG	20 (44)	17 (28)		
	GT+TT	25 (56)	28 (62)		>0.05
**RS17300539**		**Unadjusted model**		**Adjusted model** a	
		**OR (95%CI)**	**p**	**OR (95%CI)**	**p**
Additive model regression	GG	1		1	
	GA	1.281 (0.321–5.119)	0.73	1.266 (0.306–5.240)	=0.75
	AA	None	None	None	None
Dominant model regression	GG	1		1	
	GA+AA	1.281 (0.321–5.119)	0.73	1.266 (0.306–5.240)	=0.75
		**Controls (n=45)**	**PCOS (n=45)**		**p**
Genotype frequency comparison	GG	41 (91)	40 (89)		
	GA	4 (9)	5 (11)		>0.05
	AA	None	None		
Allele frequency	G/A	0.96/0.04	0.94/0.06		>0.05

a Adjusted for BMI and age.

OR: odds ratio; CI: confidence interval; PCOS: polycystic ovary syndrome.

Clinical data comparisons in the case group between genotypes in both additive and dominant models were given in Table 3. No significant difference was detected among genotypes in terms of clinical data.

**Table 3:** Comparison of clinical characteristics between genotypes in the case group.

RS1501299	Additive model			p	Dominant model		p
Genotype	GG	GT	TT		GG	GT+TT	
BMI	23.6±3.69	26.1±6.77	23.8±3.3	>0.05	23.5±3.69	24.7±6.4	>0.05
Fasting glucose (mmol/L)	5.24±0.62	5.13±0.73	4.74±0.95	>0.05	5.24±0.62	5.07±0.77	>0.05
Insulin (pmol/L)	166±123	161±166	118±30	>0.05	167±123	155±154	>0.05
Testosterone (nmol/L)	1.64±0.91	1.52±0.68	1.43±0.78	>0.05	1.64±0.91	1.51±0.69	>0.05
Estradiol (pmol/L)	298±190	253±240	347±231	>0.05	298±190	267±239	>0.05
Progesterone (ng/mL) (nmol/L)	12.0±14.8	7.1±8.76	14.2±14.3	>0.05	11.0±14.7	8.18±9.96	>0.05
FSH (IU/L)	7.85±3.72	8.24±4.21	7.4±3.32	>0.05	7.85±3.72	8.11±4.07	>0.05
LH (IU/L)	10.4±6.16	12.8±15.2	6.87±2.55	>0.05	10.4±6.16	11.94±14.31	>0.05
Prolactin (μg/L)	12.8±7.15	15.2±11.5	10.7±4.71	>0.05	12.85±7.15	14.53±10.85	>0.05
TSH (IU/L)	2.99±1.63	2.49±1.83	2.1±1.26	>0.05	2.99±1.63	2.44±1.76	**=0.031**
**RS17300539**	**Additive model**			**p**	**Dominant model**		**p**
**Genotype**	**GG**	**GA**	**AA**		**GG**	**GA+AA**	
BMI	23.6±3.69	26.1±6.77	N.A.	>0.05	23.6±3.69	26.1±6.77	>0.05
Fasting glucose (mmol/L)	5.16±0.74	4.99±0.43	N.A.	>0.05	5.16±0.74	4.99±0.43	>0.05
Insulin (pmol/L)	164±147	120±81	N.A.	>0.05	164±147	120±81	>0.05
Testosterone (nmol/L)	1.57±0.81	1.50±0.58	N.A.	>0.05	1.57±0.81	1.50±0.58	>0.05
Estradiol (pmol/L)	289±226	202±133	N.A.	>0.05	289±226	202±133	>0.05
Progesterone (ng/mL) (nmol/L)	9.98±12.7	7.48±7.35	N.A.	>0.05	9.98±12.7	7.48±7.35	>0.05
FSH (IU/L)	7.96±3.99	8.45±3.38	N.A.	>0.05	7.96±3.99	8.45±3.38	>0.05
LH (IU/L)	11.2±12.0	12.0±7.47	N.A.	>0.05	11.2±12.0	12.0±7.47	>0.05
Prolactin (μg/L)	13.6±9.33	16.0±11.2	N.A.	>0.05	13.6±9.33	16.0±11.2	>0.05
TSH (IU/L)	2.61±1.55	3.13±2.93	N.A.	>0.05	2.61±1.55	3.13±2.93	>0.05

Fallowing ±symbol standart deviation values were given. BMI: body mass index; FSH: follicle-stimulating hormone; LH: luteinizing hormone; TSH: thyroid-stimulating hormone. The bold value indicates statistically significant values.

## DISCUSSION

In a Chinese cohort of 207 patients and 192 controls, the homozygous polymorphic genotype (TT) frequency of rs1501299 was significantly higher in controls than in patients, showing a protective effect of the polymorphic (TT) genotype against PCOS^3^. Similarly, in a Chinese family-based study, the polymorphic homozygous genotype (TT) of rs1501299 was found to be protective against PCOS (p<0.05)^4^. In Polish women, the rs1501299 T allele was found to be associated with a protective effect against PCOS^5^. Similarly, in a cohort from Jordan, the T allele was found to be protective against PCOS (p=0.0064)^6^. These results are consistent despite being obtained from different populations and are surprising because they highlight the protective effect of polymorphism.

In a recent review, it was reported that rs1501299 was found to be significantly associated with PCOS under the heterozygous model^7^. Ranjzad et al. didn't observe any association between PCOS and rs1501299 in Iranian women^8^. On the other hand, Mushtaq et al.^9^ found a strong association between rs1501299 and PCOS. However, similar to the results of Czeczuga-Semeniuk et al.^5^ in Polish women, we didn't find any association between rs1501299 and PCOS. The inconsistencies between prior and our study findings may arise from heterogeneity of PCOS, ethnic, and methodological differences. Similarly, in all the mentioned studies, the rs1501299 polymorphic allele and genotype frequencies differ widely, reflecting the homogeneity among populations. According to the review of Xian et al.^10^ the TT genotype (homozygous polymorphic) frequency of rs1501299 in case groups of eight studies varies from 0.056 to 0.15, showing a notable frequency variation among ethnic groups. Our cohort displayed a frequency of 0.07 in the PCOS group. Liu et al.^11^ reported that in Caucasian and East Asian populations, the rs1501299 polymorphism was found to be associated with PCOS under a dominant model. This finding is stronger than ours since the mentioned review analyzed eighteen studies whose sample sizes were bigger and population coverage is bigger, covering Jordon, Poland, Spain, Finland, Korea, India, the United States, Brazil, Iran, Spain, Greece, Japan, and China. Similarly, in the Pakistani population, Mushtaq et al. found that rs1501299 was associated with PCOS under both genotypic and allelic models^9^.

Sun et al.^12^ reported that in the Chinese Han population, the GA and AA genotypes of rs17300539 are associated with PCOS. Similarly, Zhang et al.^13^ found an association between rs17300539 and PCOS. Contrary to these findings, we didn't observe any association between PCOS and rs17300539 in our cohort. Also, we couldn't find any BMI difference between the PCOS and the control group regarding rs17300539 (p>0.05). Dated to a 2021, in a digital weight loss program, Sinha et al^14^ reported that subjects who had polymorphic alleles of rs17300539 lost more weight compared to those with the homozygous normal genotype. In the same study, it was also suggested that each G allele added more weight loss^14^. This result is eye-catching since other studies regarding risk alleles of rs17300539 focused on overweight participants and body mass index (BMI) instead of weight loss. Losing more weight in subjects who have risk alleles of rs17300539 indicates a stronger effect of the polymorphism on BMI, following an interpretation of the polymorphism's effect on PCOS that is more than expected before.

BMI stratification and comparison between groups and genotypes gain more importance when investigating associations with polymorphisms related to adipose tissue, which is lacking in this study as a limitation. Also, an important limitation of our study is that we couldn't measure serum adiponectin levels of participants due to the lack of this test in our hospital and laboratory; therefore, we are not able to compare this parameter between groups, as well as between genotypes, and prior studies. However, Nambiar et al.^15^ commented that serum adiponectin level is more subject to BMI than PCOS disease itself.

In order to account for the effects of the homeostatic model assessment for ınsulin resistance (HOMA-IR) as a metabolic confounder, we performed HOMA-IR-adjusted logistic regression under additive, dominant, and recessive models. However, we didn't detect any significant effect of HOMA-IR in all three models on having PCOS (p>0.05, all).

Hormonal imbalance, especially hyperandrogenism, may or may not exist with PCOS. Our case group displayed a mean testosterone level of the PCOS group that was significantly higher than that of the control group (p<0.001). Ramezani Tehrani et al.^16^ and Nambiar et al.^15^ were also reported that the testosterone level of PCOS groups was higher than that of controls regarding 1501299 (p<0.05). However, Ezzidi et al.^17^ showed that the testosterone level of controls was significantly higher than that of the PCOS group (p<0.001).

Ezzidi et al. also reported that the mean progesterone level of PCOS groups was significantly higher than that of controls (p<0.001). Since the PCOS condition generally displays normal levels of estrogen, these contradictory results may arise from distinct designs of the mentioned studies as well as from PCOS heterogeneity and/or methodological differences.

### Power analysis

For rs1501299, San Millán et al.^18^ included 72 cases and 42 control patients, achieving a power value of 0.15 with an effect size of 0.085 in their study. Escobar-Morreale et al.^19,20^ included 74 cases and 40 controls and achieved a 0.06 power value with an effect size of 0.02. These studies were done based on allele frequencies from prior studies. When we calculated power and effect size from Yoshihara et al.^21^ with 59 cases and 97 controls, we had a power value of 0.72 and an effect size of 0.20. We also calculated a power value of 0.06 and an effect size of 0.02 with higher sample sizes, 100 cases and 140 controls from Xian et al.^10^ For rs17300539, 204 cases and 150 controls, Mushtaq et al.^9^ achieved a power score of 0.62 and an effect size of 0.12, while Zhang et al.^22^ with 207 cases and 192 controls, achieved a power score of 0.96 and an effect size of 0.19.

Our power score was 0.05 with an effect size of 0.02 for 1501299, and 0.13 with an effect size of 0.08 for rs17300539. Thus, while there are power and effect size values of prior studies above and below ours, the low power and effect size values of our study must be interpreted very carefully since this is a major limitation of the study and may cause type II errors (false negatives). Soares-Jr et al.^23^ and Baracat et al.^24^ explained the phenotypic heterogeneity of PCOS very well in their articles. Also, Baba et al.^25^ explained that phenotypic heterogeneity is in excess among PCOS patients over Asian, Hispanic, Eastern, and African populations. Thus, findings of this and the mentioned studies need to be evaluated with caution of phenotypic heterogeneity. As a major limitation of this study, we couldn't measure serum adiponectin levels. However, in the following future studies, correlating serum adiponectin levels with genotypes as a functional validation will be crucial in order to assess the biological relevance of the polymorphisms beyond remaining speculative.

## CONCLUSION

Here, with this study, for the first time in the studied population, we conclude that both rs1501299 and rs17300539 of the ADIPOQ gene are not associated with PCOS.

## Data Availability

The datasets generated and/or analyzed during the current study are available from the corresponding author upon reasonable request.
